# Supplementary material for: The Quasimesenchymal Pancreatic Ductal Epithelial Cell Line PANC-1—A Useful Model to Study Clonal Heterogeneity and EMT Subtype Shifting
Source: Cancers (Basel). 2022 Apr 19;14(9):2057. doi: 10.3390/cancers14092057 (PMC9101310; doi:10.3390/cancers14092057)

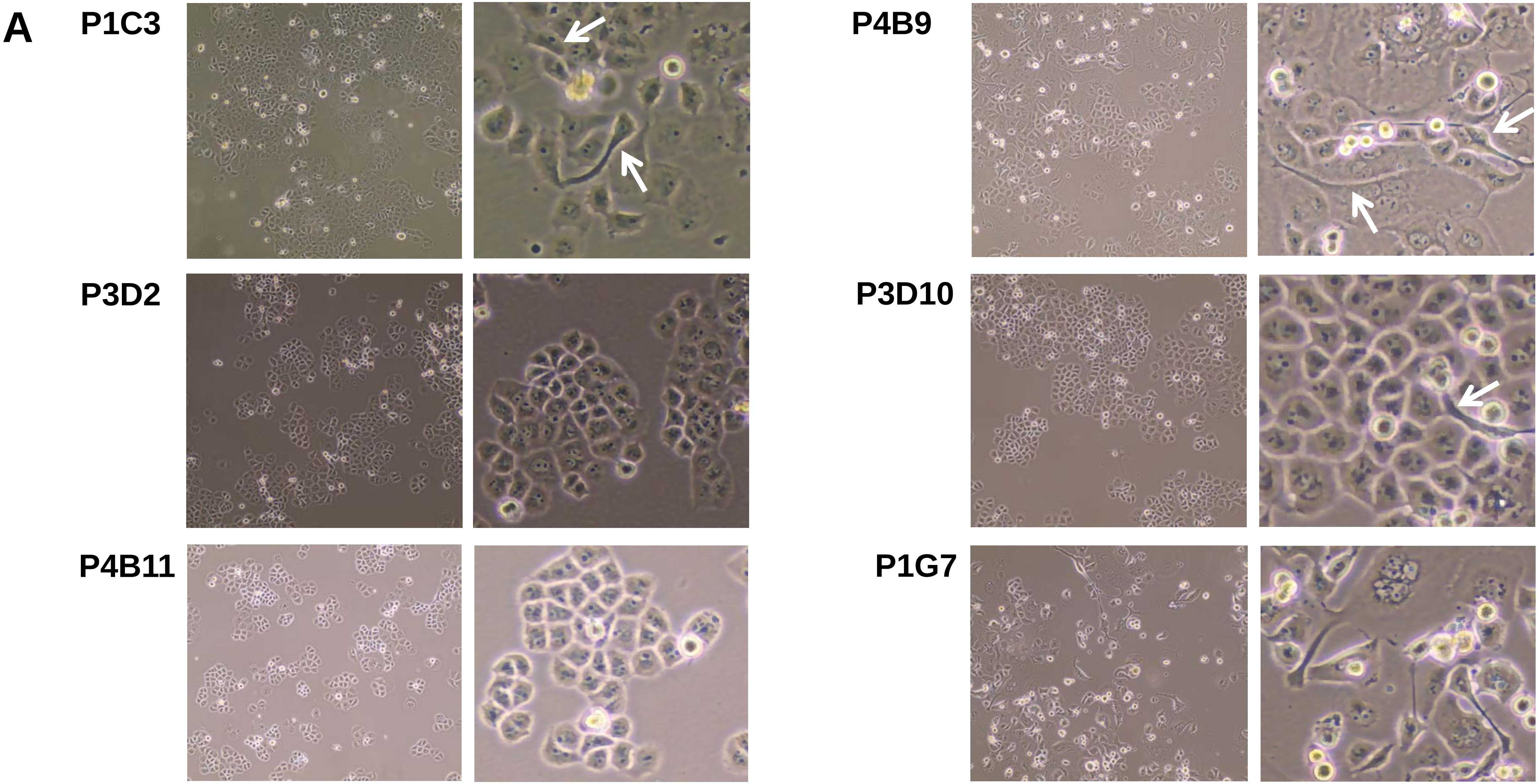

Figure S1A. Morphology and growth pattern of single cell-derived clones of PANC-1. Clonal cultures of P1C3 and P4B9 contain many spindle-shaped cells (indicated by arrows) that are mostly scattered together being indicative of an M-phenotype. Clones P4B11 and P3D2 only consist of cuboid cells with a cobblestone growth pattern consistent with an E phenotype. Clone P3D10 presents with only a few fibroblast-like cells (arrow) and may have an intermediate (E/M) phenotype. Clone P1G7 is characterized by a large fraction of cells that are either spindle-shaped or rounded up and refractive reminiscent of senescent cells. Magnification: x100 (left-hand image), x400 (right-hand image). Microscopic images of the parental cells are shown in Figure S8.

B

P3D2

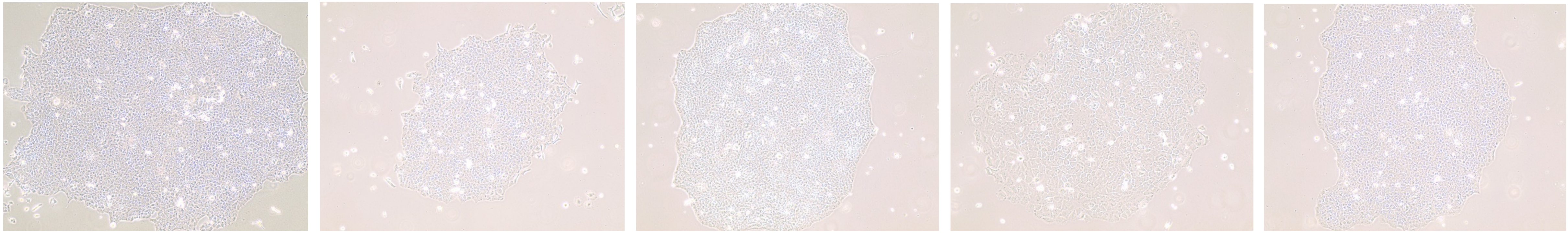

P4B11

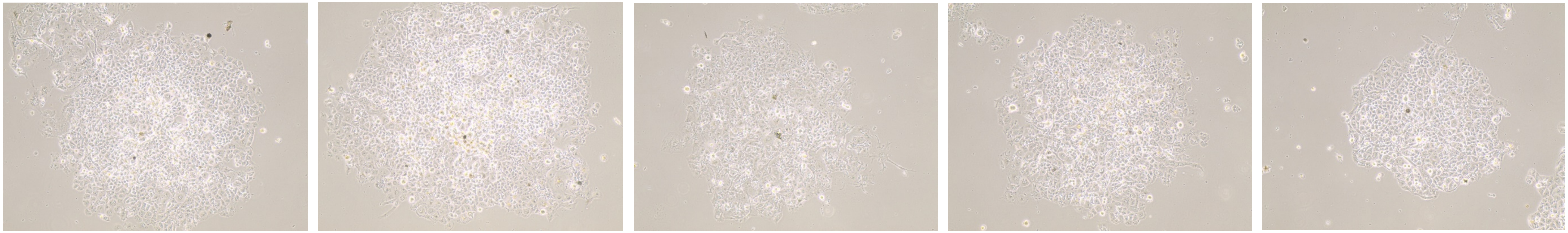

Figure S1B. Morphology and growth pattern of single cell-derived subclones of PANC-1 clones P3D2 and P4B11. These subclones were derived by limited dilution of single cell suspensions of P3D2 and P4B11. Please note the very similar growth pattern of the 5 subclones of each of the two clones. Magnification: x100. For enhanced contrast and resolution, images should be enlarged on the computer screen.

Figure S2

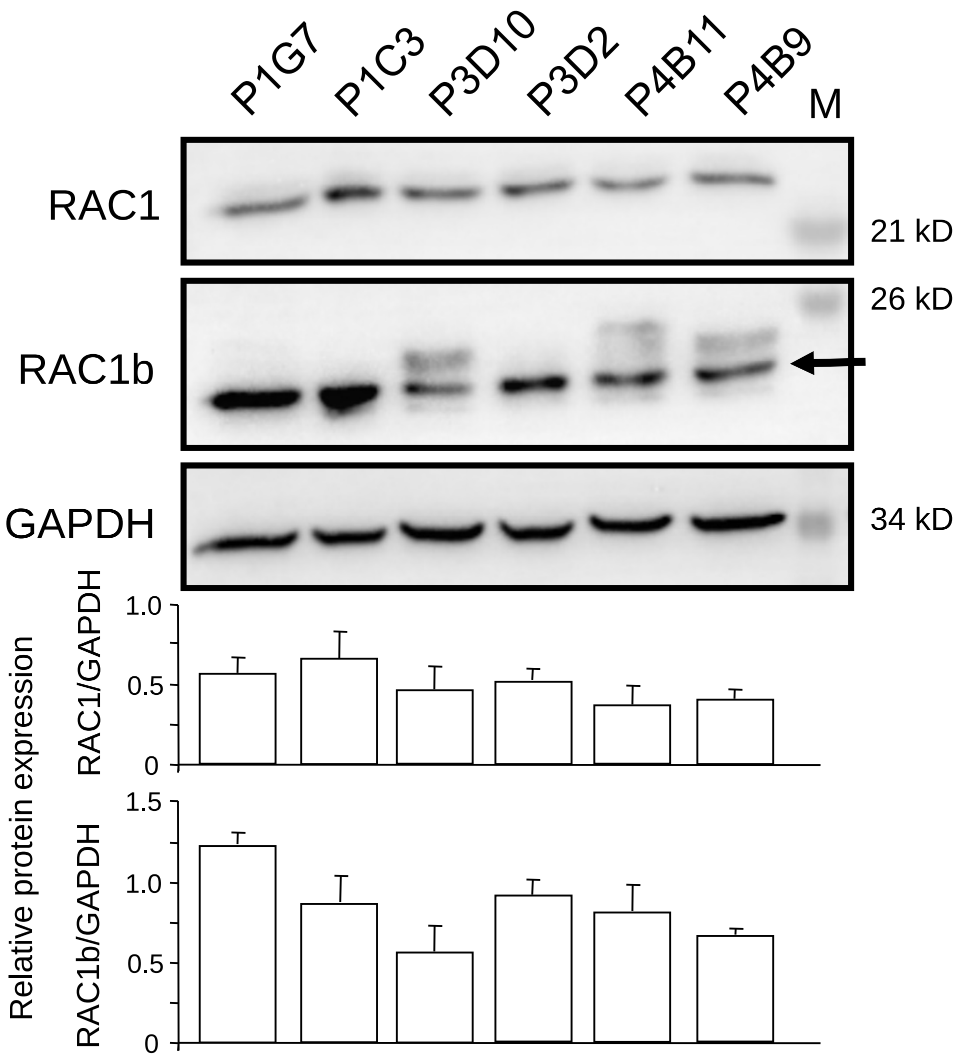

Figure S2. Quantification of RAC1 and RAC1b protein levels in PANC-1 single clones. Immunoblot analysis of RAC1, RAC1b, and GAPDH as a loading control. The graphs below the blots represent data from densitometric readings taken from a representative experiment out of three experiments performed in total (mean ± SD of triplicate wells). M, molecular weight marker

Figure S3

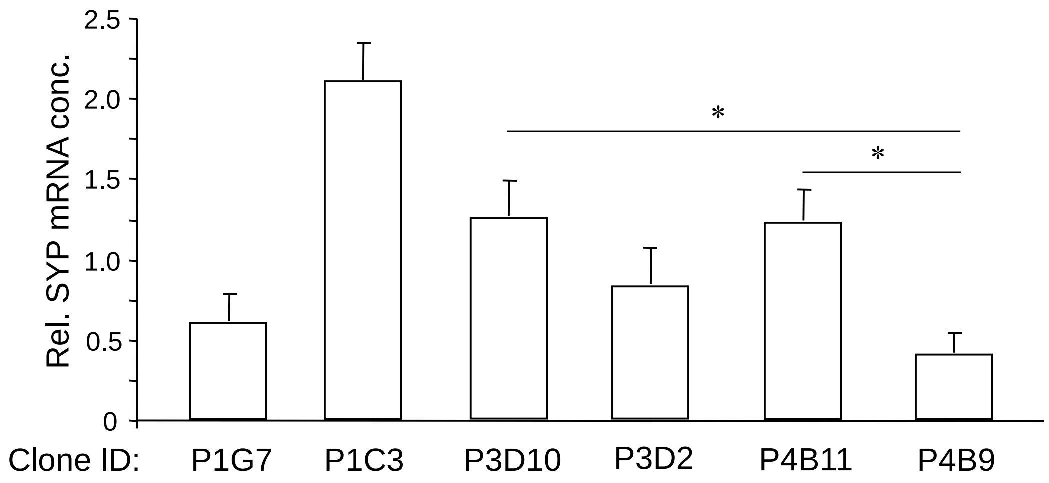

Figure S3. Expression analysis of the neuroendocrine marker SYP in the indicated PANC-1 single cell-derived clones. Cells were subjected to RNA isolation and qPCR analysis of SYP. Data shown are representative of three experiments (mean ± SD of triplicate wells). Differences between clone P1C3 and each of the other clones are significant ($p < 0.05$, two-tailed unpaired Student's t-test) as are differences between P4B9 and P3D10, or P4B9 and P4B11 (indicated by asterisks).

Figure S4

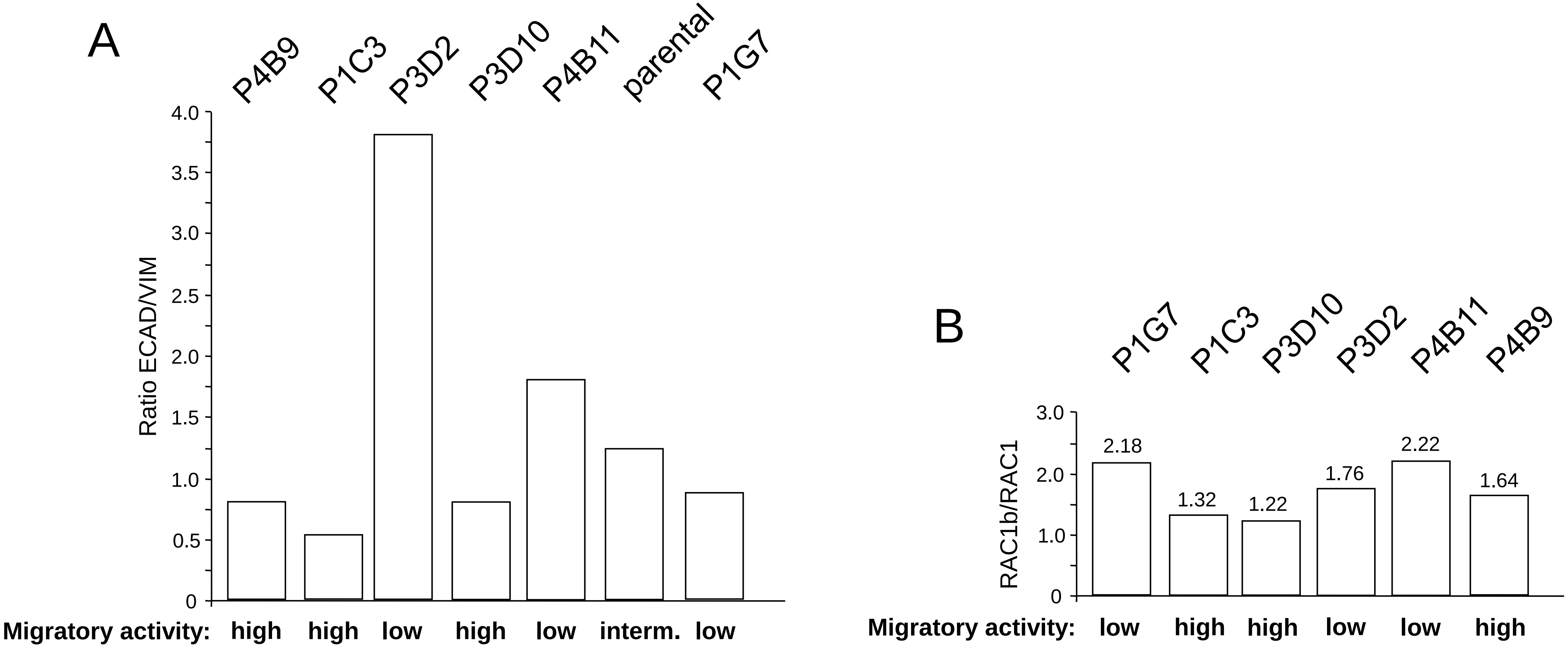

Figure S4. Correlation of the ECAD/VIM and RAC1b/RAC1 ratios with migratory activities in six individual PANC-1 clones. The ECAD/VIM ratios from Figure 1A (**A**) and the RAC1b/RAC1 ratios from Figure S2 (**B**) were allocated to the respective migratory activities of the six clones shown in Figure 2A. Their migratory activities were scored as either high (P1C3, P3D10, P4B9) or low (P1G7, P3D2, P4B11), respectively, and are indicated for each clone underneath the respective ECAD/VIM (**A**) and RAC1b/RAC1 (**B**) ratios; interm., intermediate.

Figure S5

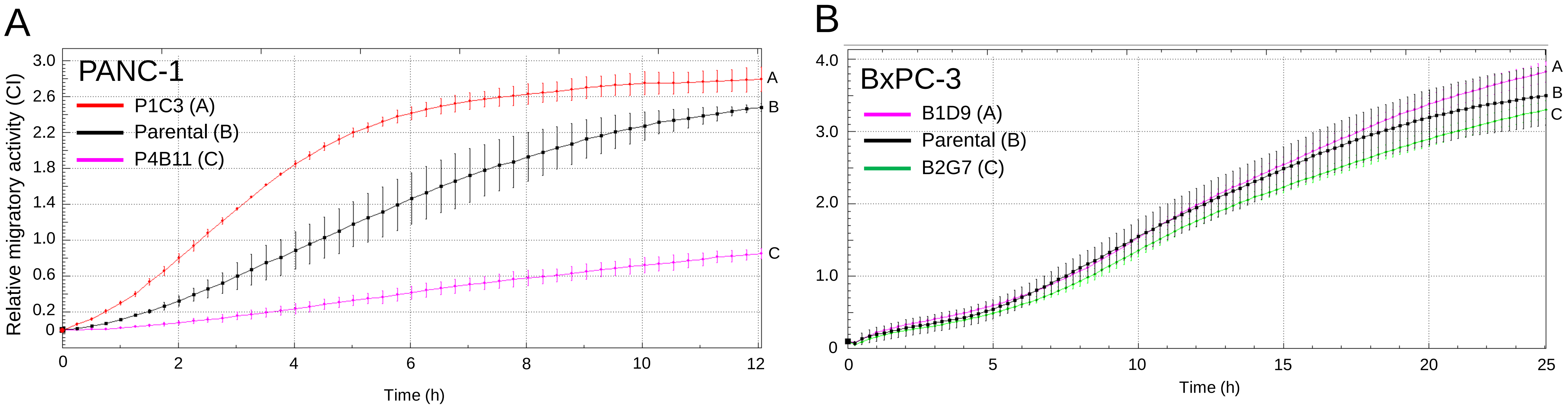

Figure S5. Migratory activity of parental PANC-1 or BxPC-3 cells compared to two single cell-derived clones. Real-time cell migration assays were performed with parental cells of PANC-1 (**A**) or BxPC-3 (**B**) and the indicated clones as outlined in the legend to Figure 2 and the Methods section. Data in (A) and (B) are the mean ± SD of quadruplicate wells and are representative of three assays.

Figure S6

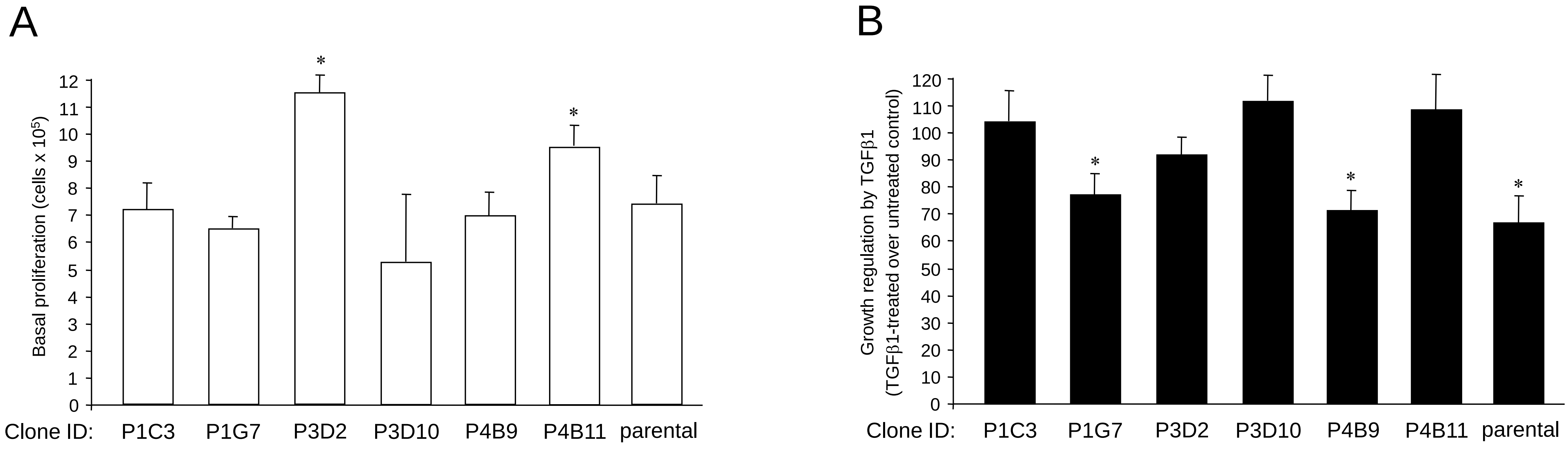

Figure S6. Proliferative responses of individual PANC-1 clones under basal conditions and after stimulation with TGFβ1 as determined by cell counting assay. Following seeding (10,000 cells/12-well), the indicated PANC-1 clones and parental cells were incubated in standard growth medium (containing 10% FCS but no TGFβ1) for 72 h (**A**), or were treated for 72 h with or without TGFβ1 (5 ng/ ml) in standard growth medium (**B**) prior to lifting and cell counting with a Neubauer chamber. Data in (A) and (B) are the mean ± SD of three independent assays. In (A), differences are significant between P3D2 and all other clones, and between P4B11 and all other clones except P3D10 (indicated by asterisks). Data in (B) are displayed as percentage of TGFβ1-treated over control cells. Control cells were set arbitrarily at 100%. The asterisks (*) indicate significant differences between TGFβ1-treated and untreated control cells.

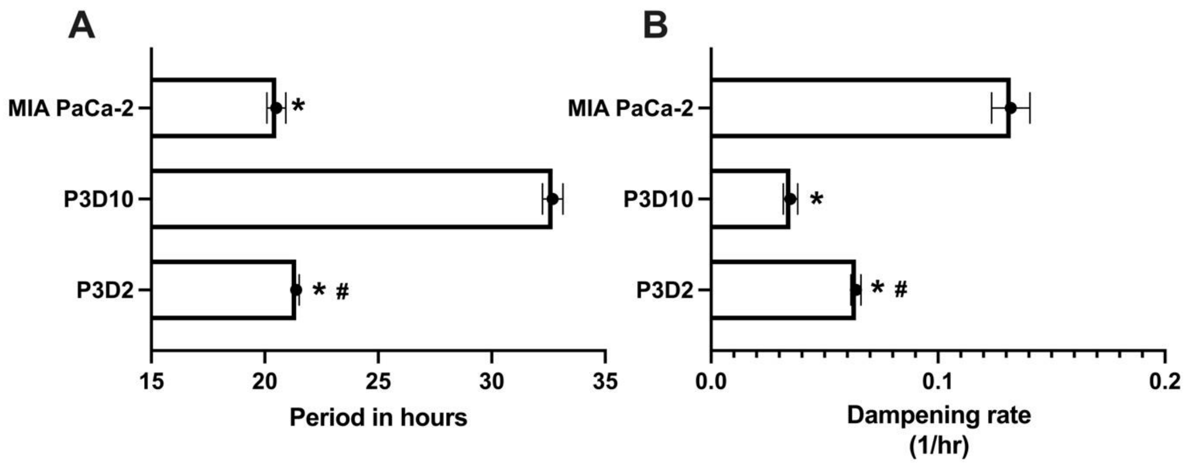

Figure S7. Rhythmic parameters evaluation by CircaSingle. (**A**) Period and (**B**) dampening rate are shown as mean ± SD comprised of n – 6 to 8 samples per group. In (A), * represents a difference between PANC-1 clone P3D10 versus the remaining PANC-1 clones. # shows a difference between PANC-1 clone P3D2 and MIA PaCa-2. In (B) * represents a difference between MIA PaCa-2 and remaining PANC-1 clones. # displays a difference between P3D2 and P3D10. * and # represent a statistical difference of < 0.0001.

Figure S8

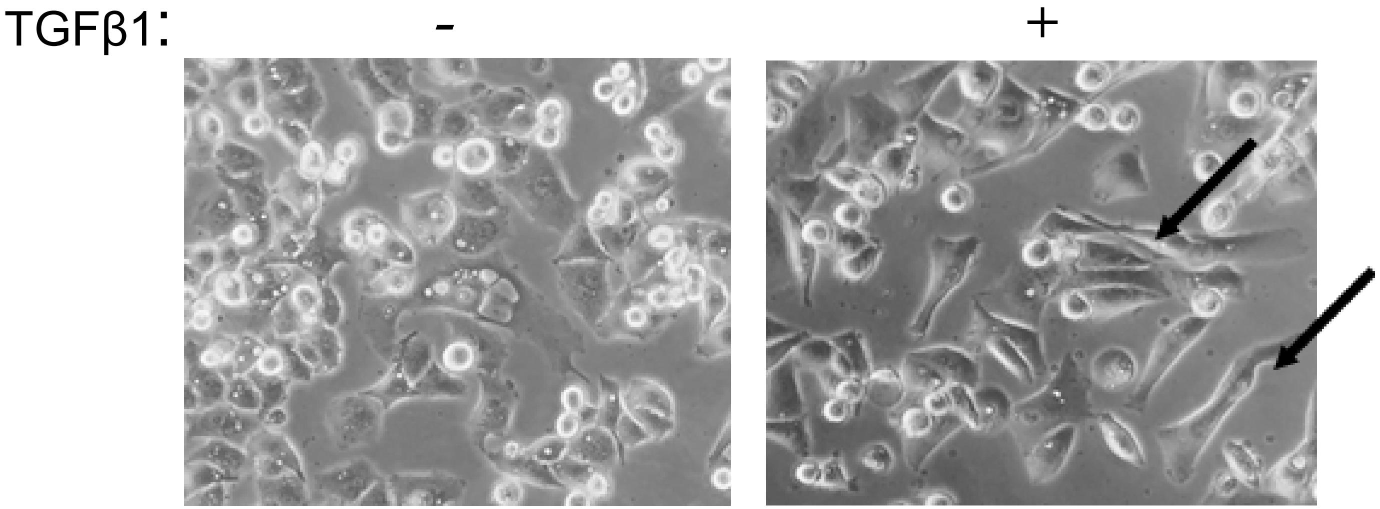

Figure S8. Morphology and growth pattern of parental PANC-1 cultures treated with TGFβ. Parental cultures of PANC-1 cells were left untreated (-), or exposed for 48 h to TGFβ1 (5 ng/ml). Please note that TGFβ1-treated cultures contain many cells with an M phenotype as evidenced by their spindle-shaped morphology and scattered growth pattern (indicated by arrows). Magnification: x200.

Figure S9

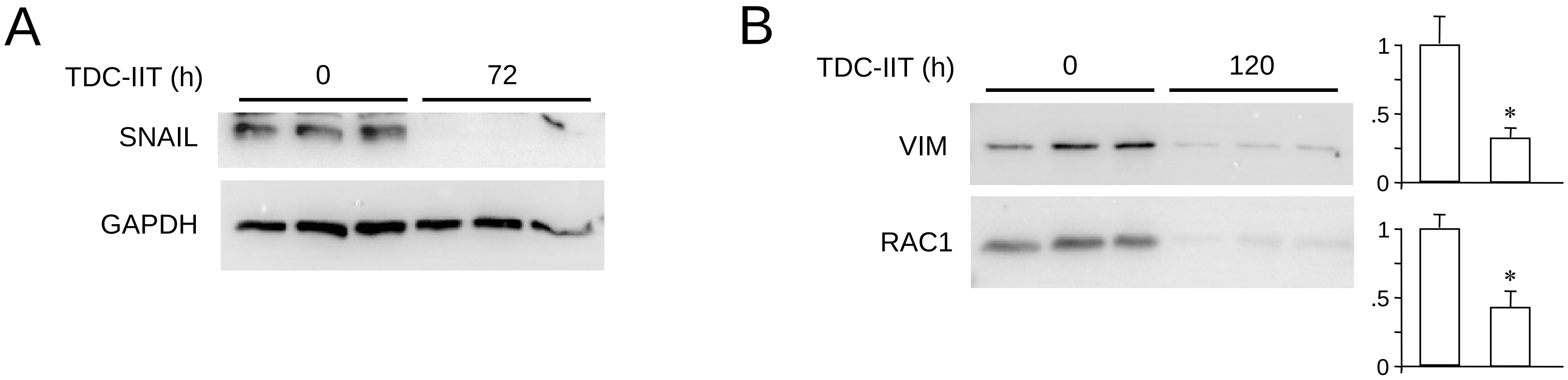

Figure S9. Immunoblot analysis of SNAIL, RAC1 and VIM in parental PANC-1 cells after treatment with IIT. (**A**) PANC-1 cells were left untreated (0 h) or were subjected to TD culture with IIT (TDC-IIT) for 72 h followed by immunoblot analysis of SNAIL, and GAPDH as a loading control. Three technical replicates were loaded for each time point. (**B**) As in (A), except that PANC-1 cells were treated for 120 h with IIT followed by immunoblotting of VIM and RAC1. The graphs to the right show data quantification based on densitometric readings of signal intensities from three technical replicates after normalization to total protein levels in each lane (means ± SD). Significant differences ($p < 0.05$) are marked by asterisks (*). The blots shown in (A) and (B) are each representative of three independent experiments.

Figure S10: uncropped blots of Figure 1

A

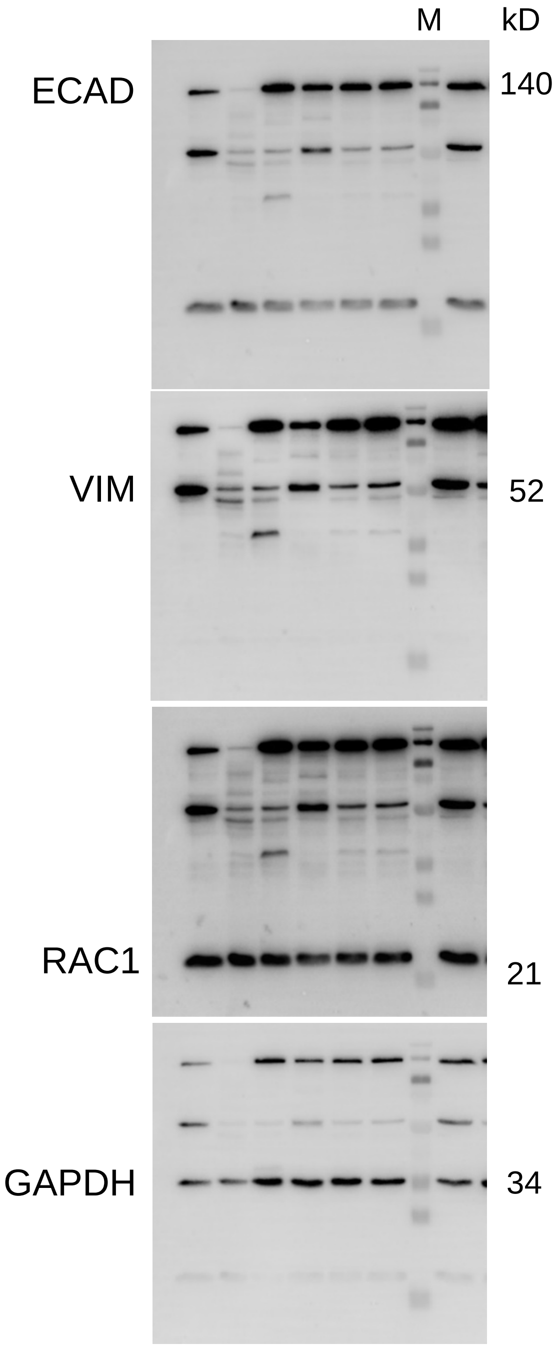

B

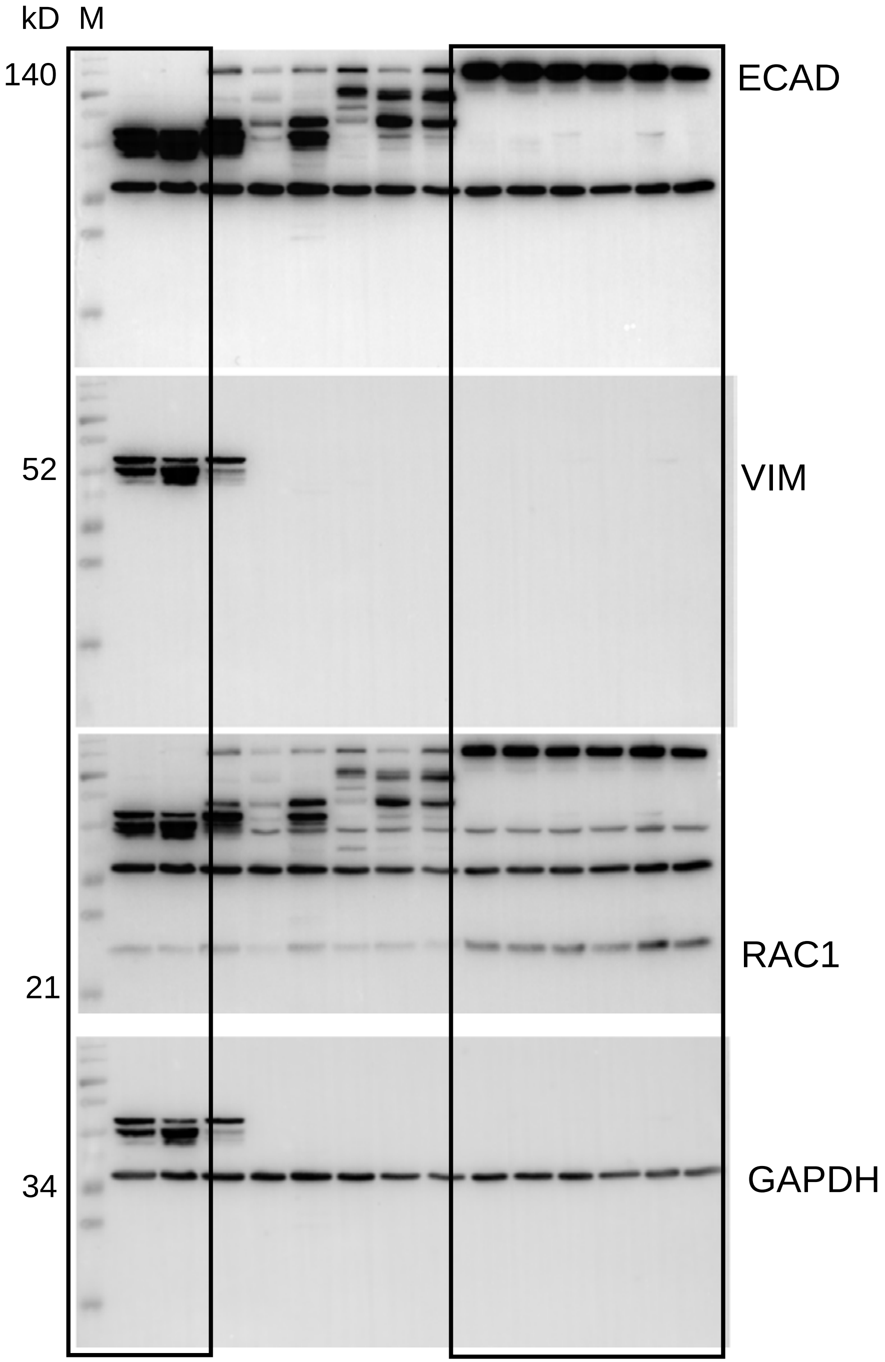

Figure legend:
M = molecular weight marker (SM1841, Fermentas/Thermo Fisher Scientific),
kD = kiloDalton

Figure S11: uncropped blots of Figure 3C

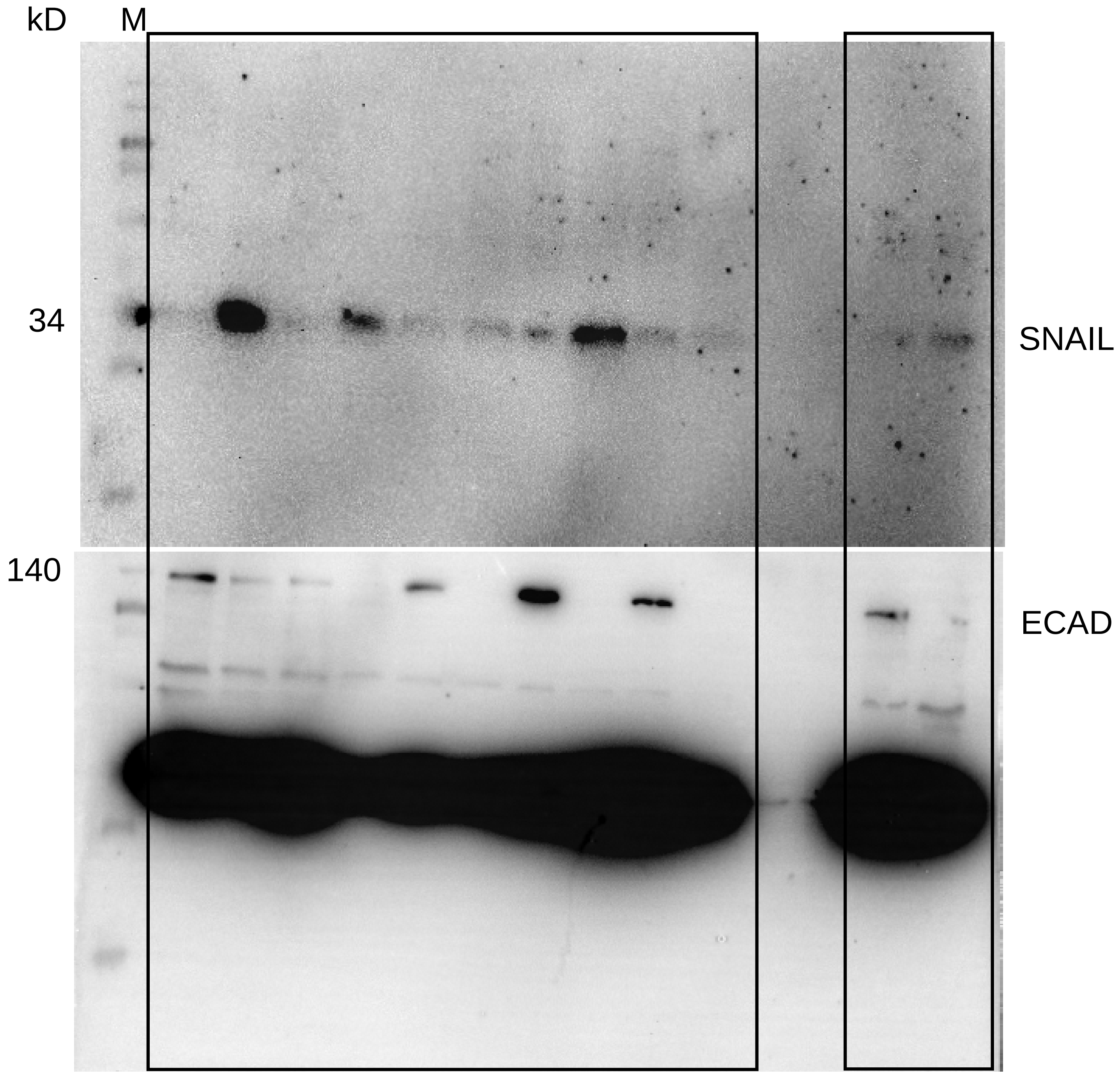

Figure S12: uncropped blots of Figure 6A+B

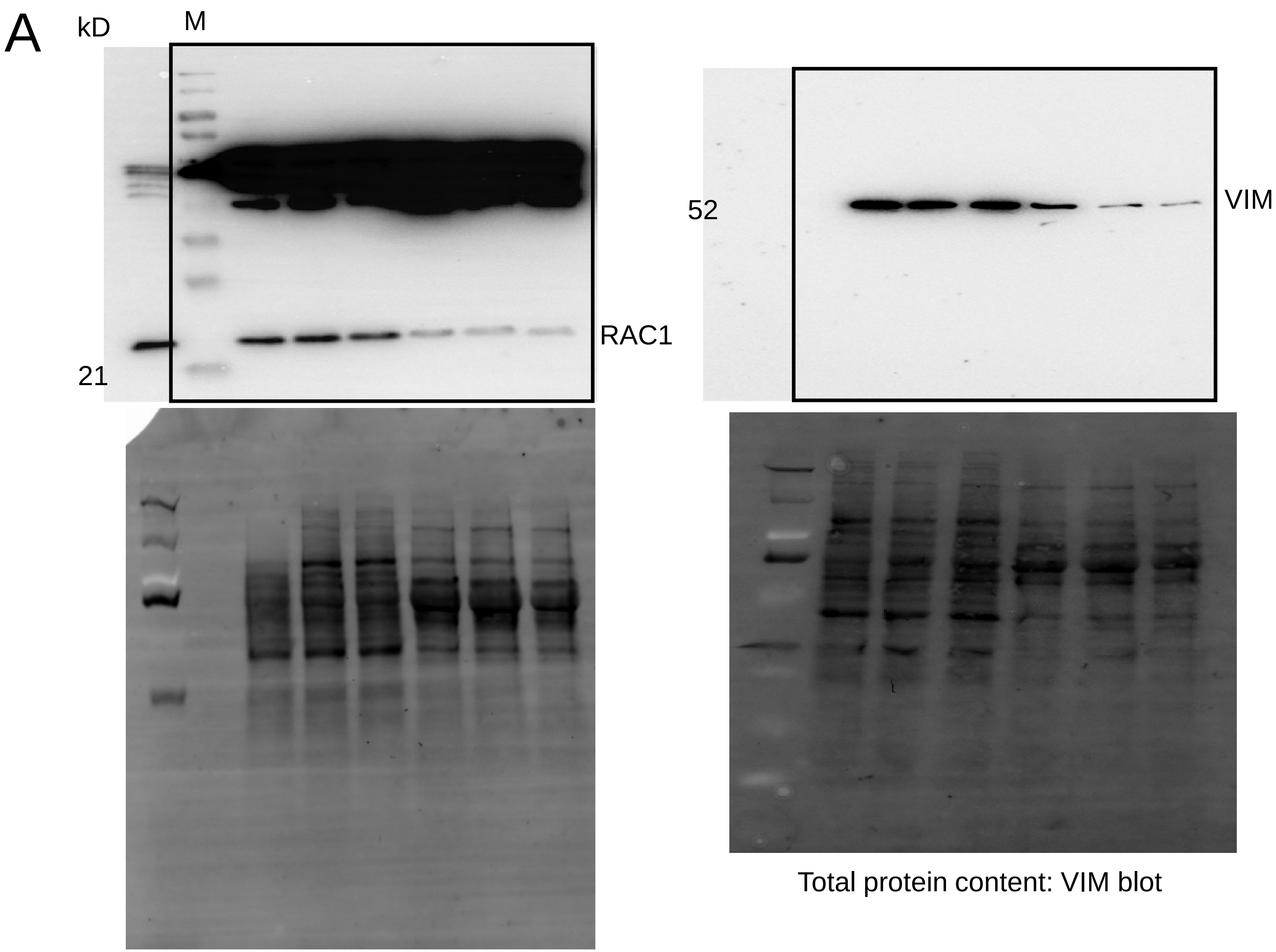

Total protein content: RAC1 blot

Total protein content: VIM blot

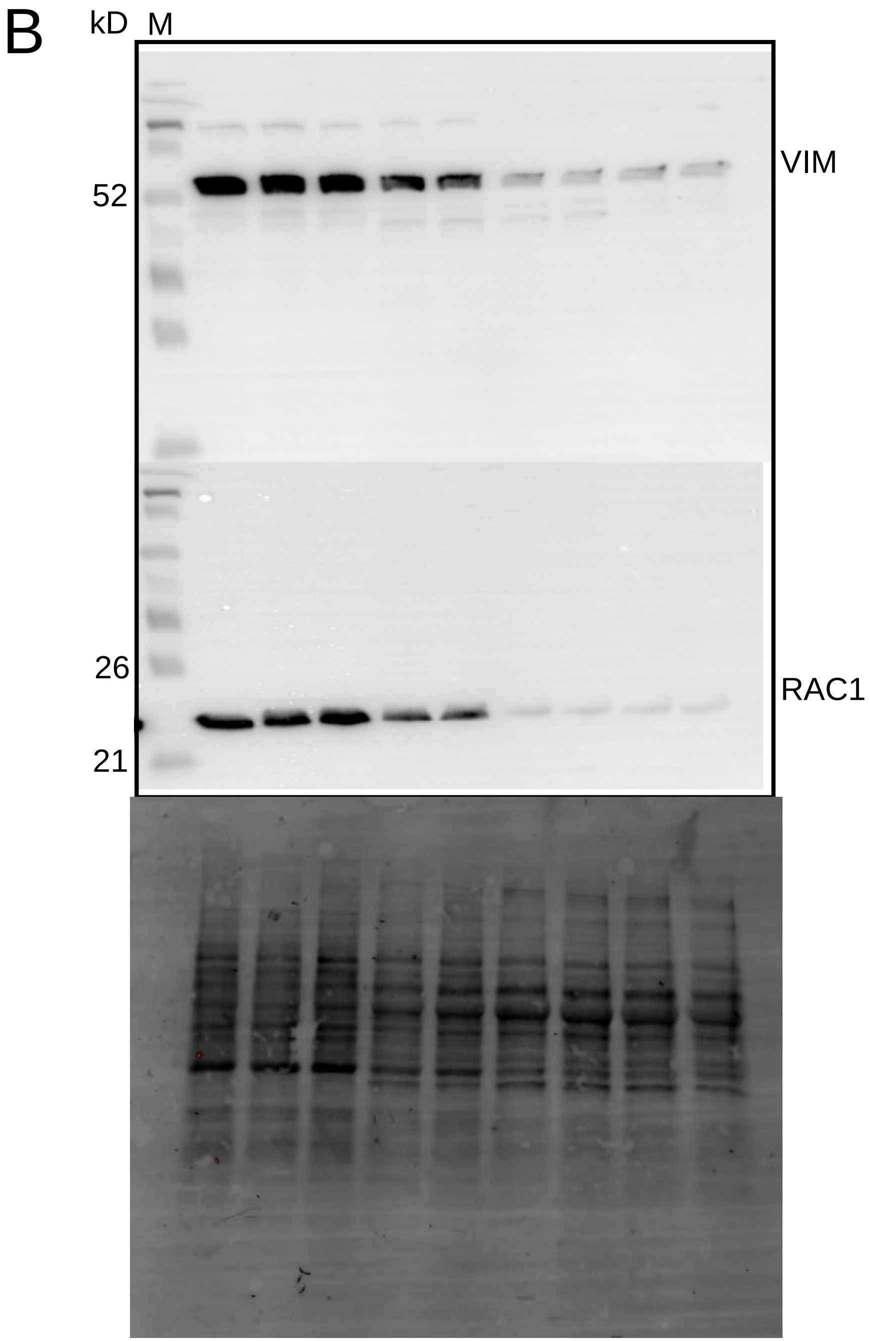

Total protein content: RAC1/VIM blot

Figure S12: uncropped blots of Figure 6C

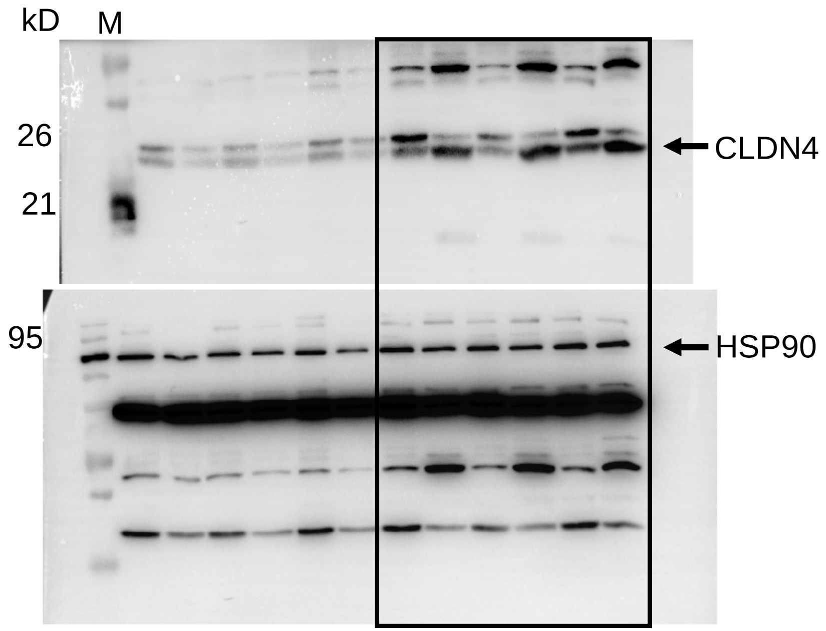

Figure S13: uncropped blots of Figure 7A+B

A

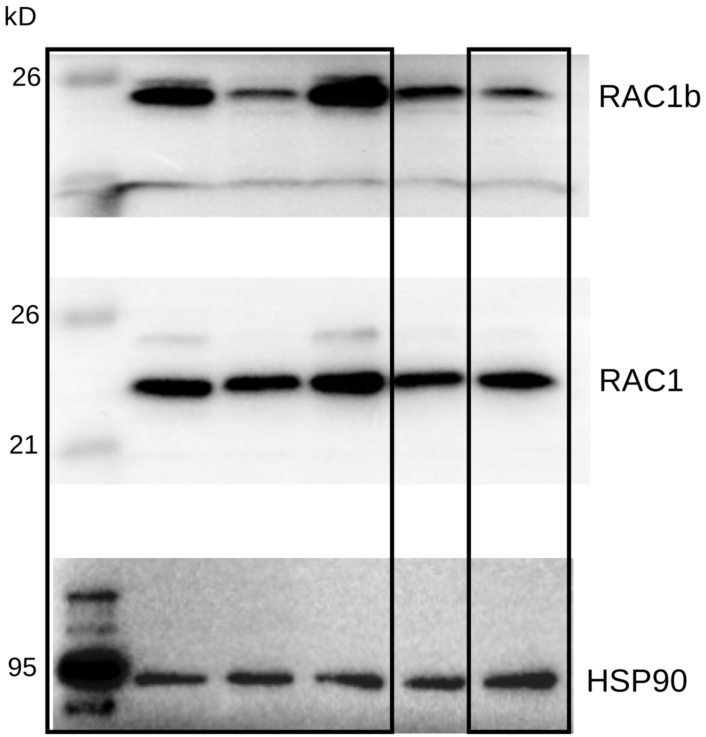

B

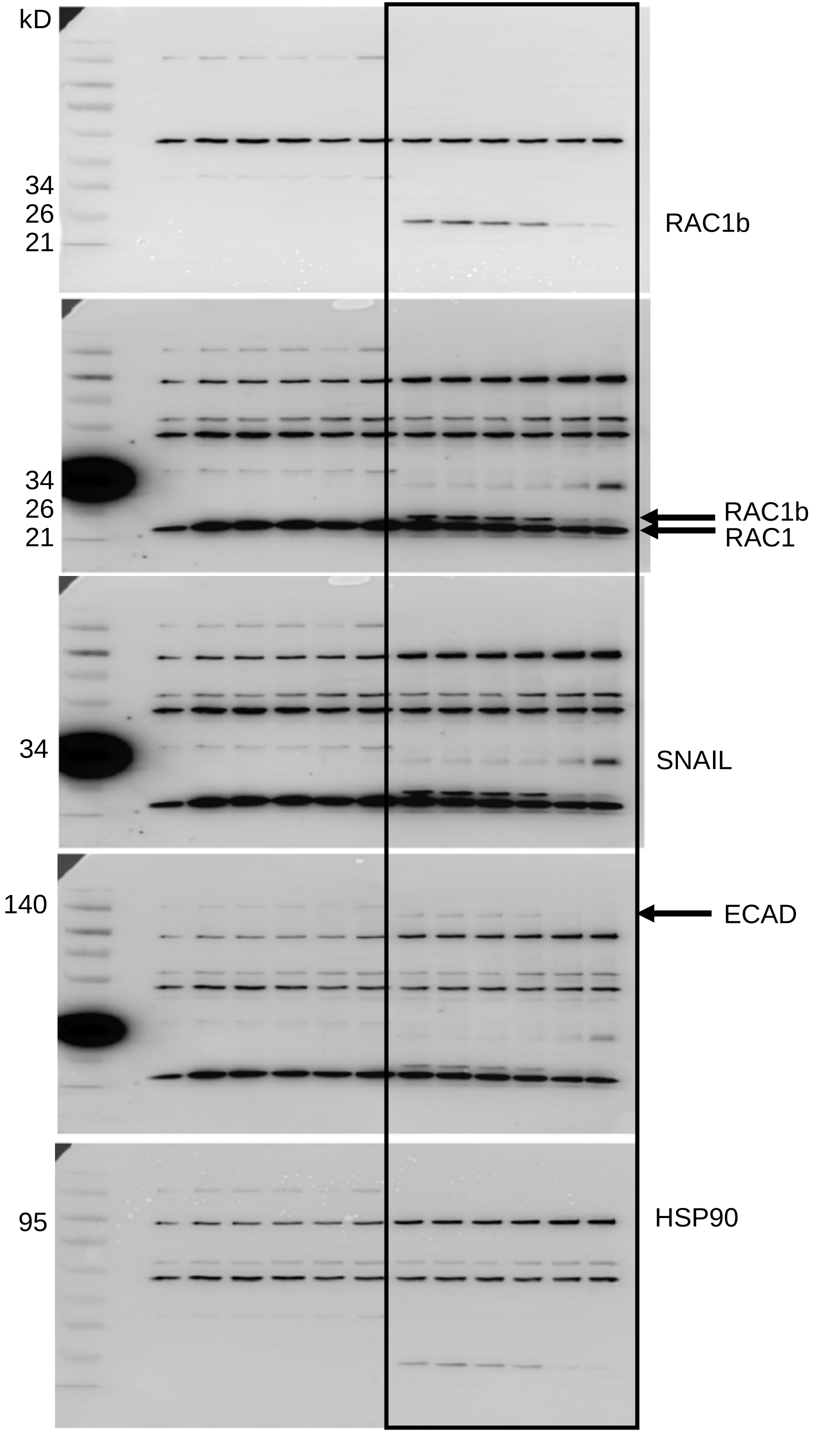

Figure S13: uncropped blots of Figure 7C+E

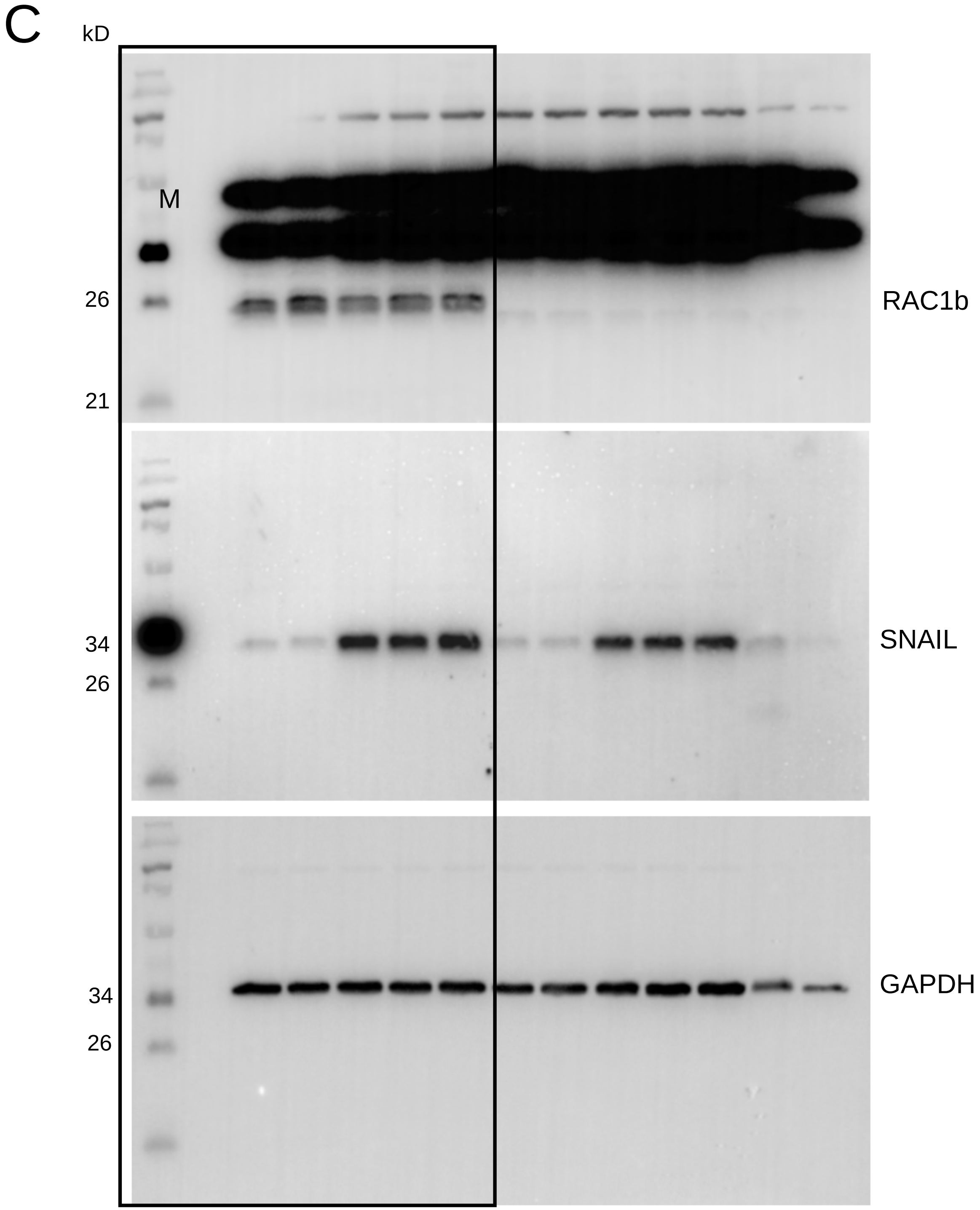

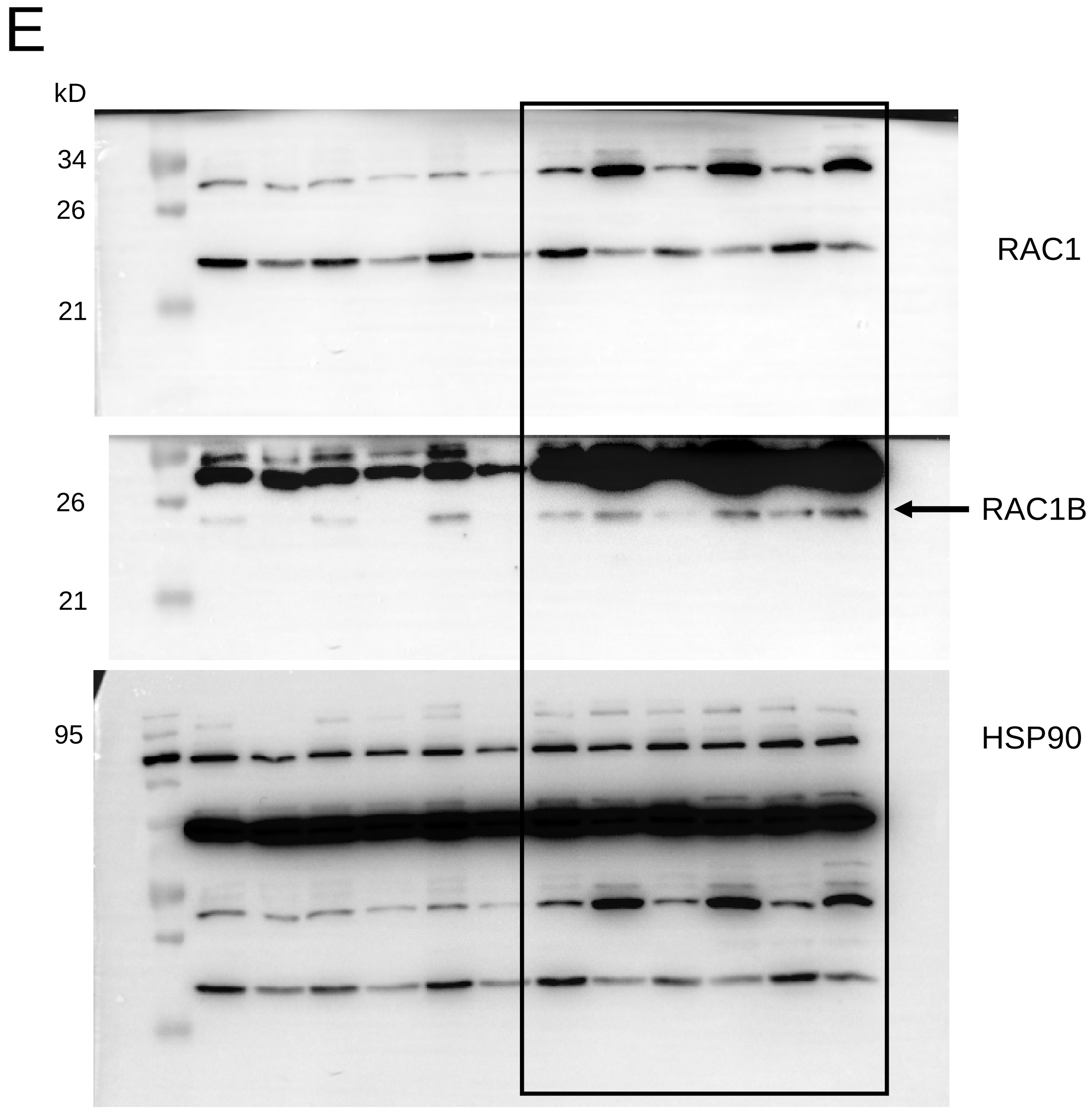

Figure S14: uncropped blots of Figure S9

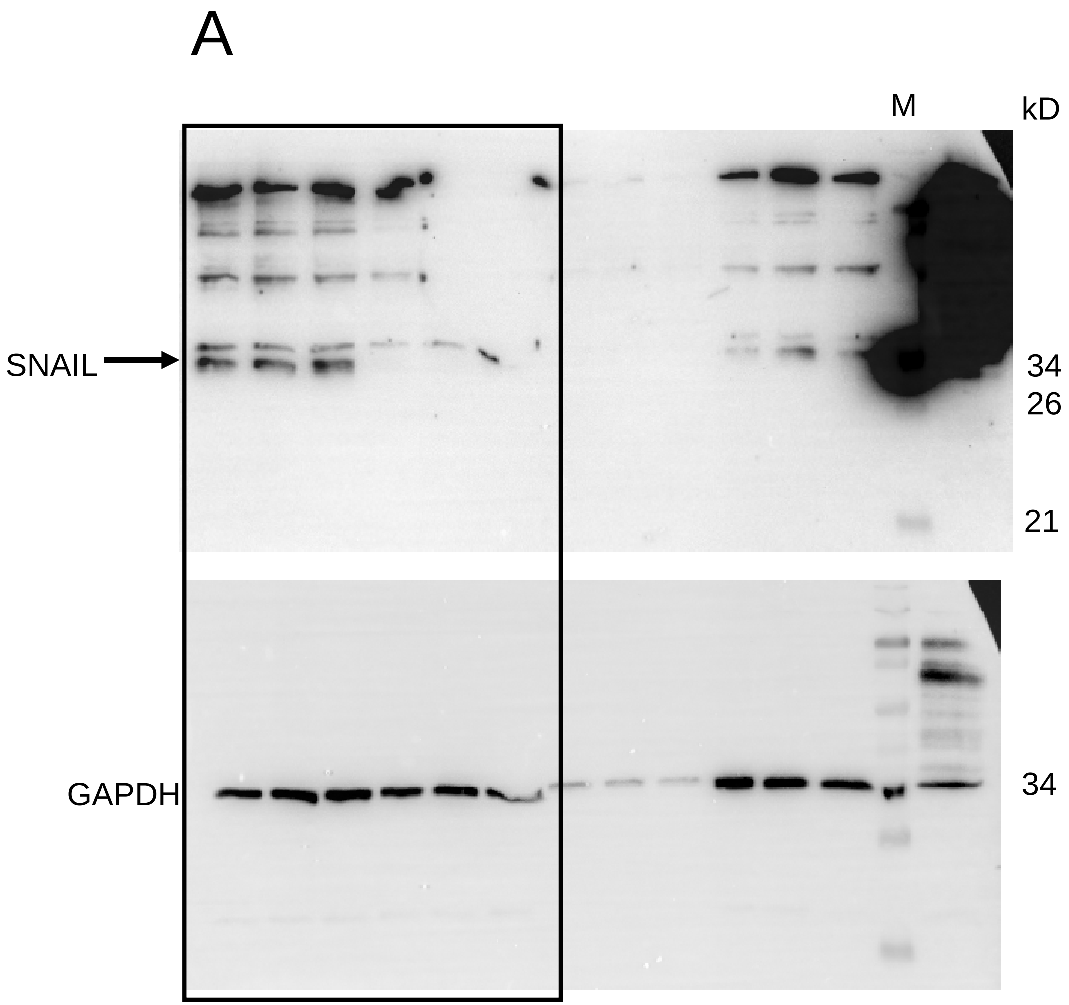

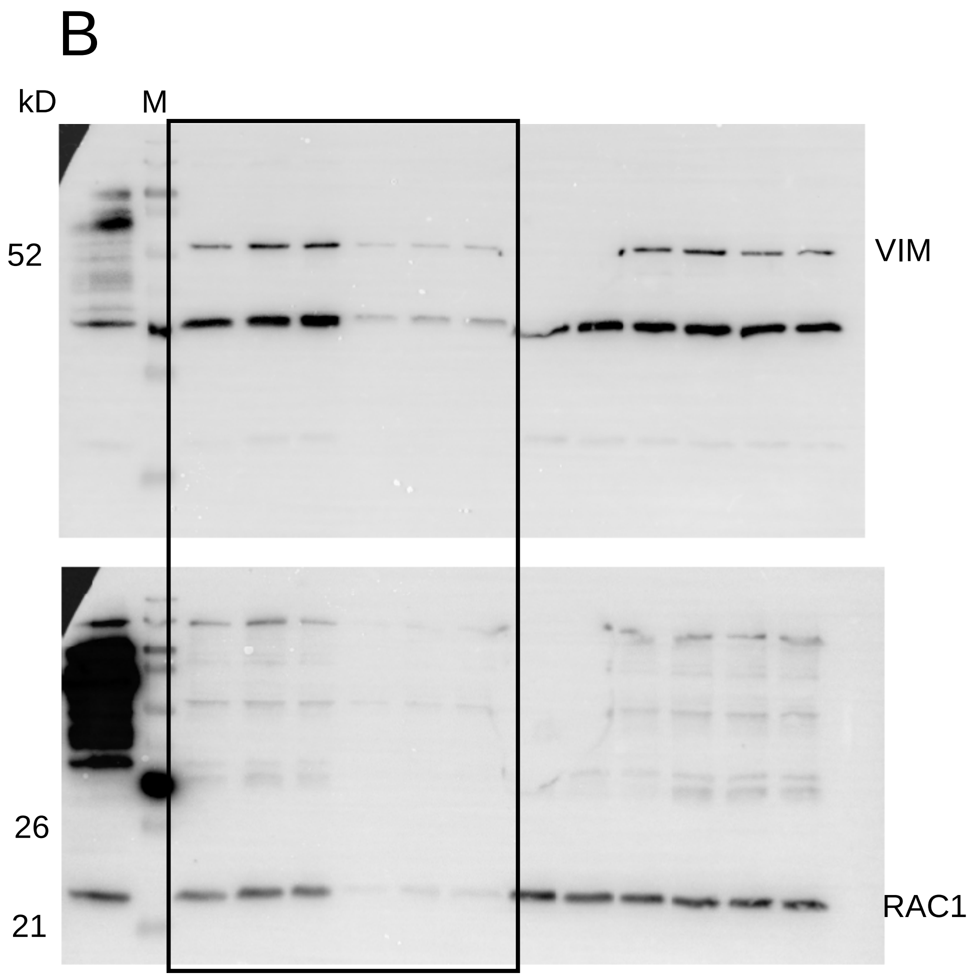

Supplement: Supplementary file 1 [file cancers-14-02057-s001.zip › cancers-1586574-supplementary.pdf]
