# Supplementary material for: The Quasimesenchymal Pancreatic Ductal Epithelial Cell Line PANC-1—A Useful Model to Study Clonal Heterogeneity and EMT Subtype Shifting
Source: Cancers (Basel). 2022 Apr 19;14(9):2057. doi: 10.3390/cancers14092057 (PMC9101310; doi:10.3390/cancers14092057)
Supplement: Supplementary file 1 [file cancers-14-02057-s001.zip › Table S1.pdf]

Table S1. Primers used for qPCR.

Gene	Forward (5'→3')	Reverse (5'→3')
BGN	CCCTCTCCAGGTCCATCCGC	GAGCTGGGTAGGTTGGGCGGG
CK19	ATGGCCGAGCAGAACCGGAA	CCATGAGCCGCTGGTACTCC
CLDN7	AGCTGCAAAATGTACGACTCG	GGAGACCACCATTAGGGCTC
ECAD	TCTTCCCCGCCCTGCCAATC	GCCTCTCTCGAGTCCCCTAG
EPCAM	AATCGTCAATGCCAGTGTACTT	TCTCATCGCAGTCAGGATCATAA
GAPDH	TTGCCATCAATGACCCCTTCA	CGCCCCACTTGATTTTGGA
GRHL2	GGGGAAGAGCAACGAGTGG	GCGCTGGTCGTCTTTGAGA
INS	GCAGCCTTTGTGAACCAACAC	CCCCGCACACTAGGTAGAGA
NEUROG3	GGCGCTGACTCAAACGCTGC	AAGCCAGACTGCCTGGGCTC
OVOL2	ACAGGCATTCGTCCCTACAAA	CGCTGCTTATAGGCATACTGC
RAC1	ACCATGCAGGCCATCAAGTGTGTGG	TTACAACAGCAGGCATTTTCTCTTC
RAC1b	GGAGAAACGTACGGTAAGGATATAACC	GGCAATCGGCTTGTCTTTGCCC
SERPINE1	CTTCTTCAGGCTGTTCCGGAGC	GGGTCAGGGTTCCATCACTTGG
SNAI1	CTGCTCCACAAGCACCAAGAGTC	CCAGCTGCCCTCCCTCCAC
SNAI2	ATATTCGGACCCACACATTACCT	GCAAATGCTCTGTTGCAGTGA
TBP	GCTGGCCCATAGTGATCTTT	CTTCACACGCCAAGAAACAG
VIM	TGGCAGGTCTTGACCTTGAA	GGTCATCGTGATGCTGAGAA
